# Ranking’s Half-Yearly Abstract, for December

**Published:** 1851-03

**Authors:** 


### ARTICLE V.

*Ranking's Half-yearly Abstract of Medical Science, for December*, 1850.

This excellent compend of Medical Science, comes to us regularly, and is always welcome to our table. It is one of the most valuable medical publications of the times; giving a synopsis of the departments of the profession, well collated and concisely reported for each succeeding six months.

It furnishes about six hundred pages of closely printed matter annually, and is furnished by the republishers, (Lindsay & Blackiston of Philadelphia,) for the low price of one dollar and fifty cents.

